# Outcome after surgical treatment of cerebrospinal fluid leaks in spontaneous intracranial hypotension—a matter of time

**DOI:** 10.1007/s00415-021-10710-7

**Published:** 2021-07-18

**Authors:** Levin Häni, Christian Fung, Christopher Marvin Jesse, Christian Thomas Ulrich, Eike Immo Piechowiak, Jan Gralla, Andreas Raabe, Tomas Dobrocky, Jürgen Beck

**Affiliations:** 1 Department of Neurosurgery, Inselspital, Bern University Hospital, University of Bern, Freiburgstrasse, 3010 Bern, Switzerland; 2 Department of Neurosurgery, Medical Center, University of Freiburg, Freiburg, Germany; 3 Department of Neurosurgery, Lindenhofspital, Bern, Switzerland; 4 Institute of Diagnostic and Interventional Neuroradiology, Inselspital, Bern University Hospital, University of Bern, Bern, Switzerland

**Keywords:** Spontaneous intracranial hypotension, Spinal cerebrospinal fluid leak, Orthostatic headache, Low ICP syndrome

## Abstract

**Objective:**

Spinal cerebrospinal fluid (CSF) leaks cause spontaneous intracranial hypotension (SIH). Microsurgery can sufficiently seal spinal CSF leaks. Yet, some patients suffer from residual symptoms. Aim of the study was to assess predictors for favorable outcome after surgical treatment of SIH.

**Methods:**

We included consecutive patients with SIH treated surgically from January 2013 to May 2020. Subjects were surveyed by a questionnaire. Primary outcome was resolution of symptoms as rated by the patient. Secondary outcome was postoperative headache intensity on the numeric rating scale (NRS). Association between variables and outcome was assessed using univariate and multivariate regression. A cut-off value for continuous variables was calculated by a ROC analysis.

**Results:**

Sixty-nine out of 86 patients (80.2%) returned the questionnaire and were analyzed. Mean age was 46.7 years and 68.1% were female. A significant association with the primary and secondary outcome was found only for preoperative symptom duration (*p* = 0.001 and *p* < 0.001), whereby a shorter symptom duration was associated with a better outcome. Symptom duration remained a significant predictor in a multivariate model (*p* = 0.013). Neither sex, age, type of pathology, lumbar opening pressure, nor initial presentation were associated with the primary outcome. ROC analysis yielded treatment within 12 weeks as a cut-off for better outcome.

**Conclusion:**

Shorter duration of preoperative symptoms is the most powerful predictor of favorable outcome after surgical treatment of SIH. While an initial attempt of conservative treatment is justified, we advocate early definitive treatment within 12 weeks in case of persisting symptoms.

**Supplementary Information:**

The online version contains supplementary material available at 10.1007/s00415-021-10710-7.

## Introduction

Spontaneous intracranial hypotension (SIH) is an important cause of incapacitating headache. It affects primarily young and middle-aged patients, with an estimated incidence of 5/100,000 [1, 2]. The hallmark symptom is orthostatic headache, but the clinical spectrum is diverse and encompasses other orthostatic symptoms such as visual or vestibulocochlear manifestations [3]. SIH is mainly caused by a spinal cerebrospinal fluid (CSF) leak primarily located in the cervical and thoracic region [2, 4–6]. While epidural blood patching remains a commonly performed intervention for spinal CSF leaks, its response rate varies widely in the literature [7]. In refractory cases, surgical closure of the spinal CSF leak is a more definitive treatment [8, 9]. Despite definitive surgical closure of the leak, some patients suffer from residual symptoms. It remains unclear, which patient benefits most from surgical treatment and which factors negatively affect outcome.

As acknowledged by the International Headache Society in the 3rd edition of the International Classification of Headache Disorders (ICHD), and supported by our previous work, the clinical signs and symptoms associated with SIH can change over time [10, 11]. The orthostatic nature of the headache can become less obvious with increasing duration of the disease, a feature that is often accompanied by a change in CSF dynamics [11]. This evolution might represent an adaptive change, but its influence on outcome has never been investigated in a clinical setting.

The aim of the present study was to assess predictors for favorable outcome after surgical treatment of SIH. Based on our previous work, we hypothesized that a longer duration of preoperative symptoms might negatively affect outcome.

## Methods

### Standard protocol approvals, registrations, and patient consents

We conducted a retrospective, observational case–control study. We obtained approval from the local ethics committee of the canton of Bern, Switzerland, for this study (2020-00645).

### Patient population

We included all patients treated surgically for SIH at our institution between January 2013 and May 2020. We excluded patients treated for secondary CSF leakage due to previous lumbar puncture, epidural anesthesia or previous spinal surgery.

Our preoperative standardized diagnostic workup was performed as described previously [12, 13]. Spinal imaging consisted of a stepwise protocol using more invasive imaging techniques with increasing suspicion of a spinal CSF leak to ultimately localize the exact site of leakage [12, 14, 15]. If no spinal CSF leak was identified despite clear signs of SIH, a CSF-venous fistula was searched by dynamic CT-myelography or digital substraction myelography.

### Surgical procedure

Surgical closure of spinal CSF leaks was performed under general anesthesia using a posterior approach with continuous intraoperative neuromonitoring as described previously [8, 9]. Through a midline incision, we performed a interlaminar fenestration or hemilaminotomy under the microscope centered on the suspected site of CSF leakage. After removal of the yellow ligament, the dura was inspected. For CSF leaks situated on the lateral aspect of the dura, direct extradural closure was usually feasible. Prolapsing arachnoid in the axilla of the nerve root was reduced, the dura sutured and augmented with an extradural wrap using TachoSil® (Takeda) or Duragen® (Integra). In contrary, for CSF leaks on the anterior dura, a dorsal durotomy and spinal cord release maneuver was performed. After cutting of the dentate ligament, the spinal cord was gently rotated using the cut ends as handles. After identification of the site of leakage, microspurs penetrating the anterior dura were removed when present, and the dural edges sutured using monofilament sutures. Intra- and extradural augmentation using dural substitutes, glue or muscle patches was done additionally at the surgeon’s discretion.

### Data analysis

Subjects were surveyed by a purposely designed questionnaire at a mean of 2.1 years after treatment. The questionnaire contained items pertaining the quality, intensity and duration of preoperative symptoms as well as outcomes variables. Patients were asked about preoperative headache intensity on the NRS, onset of headache within 5 min in the upright position, presence of visual disturbances, presence of auditory disturbances, evolution of headache into a less intense or less orthostatic character over time, duration of preoperative symptoms, application of a preoperative epidural blood patch and occurrence of postoperative rebound intracranial hypertension. In case of persistent symptoms, patients were questioned about the quality and nature of these symptoms as well as resumption of work status. The questionnaire was sent by mail to all patients fulfilling the inclusion criteria mentioned above. If no reply was received, we tried to contact patients by phone and ask them to fill out a digital version of the questionnaire. If we were not able to obtain a response despite three attempts to reach the patient by phone, the data were considered missing and excluded from the analysis. The questionnaire was available in German and in French.

Primary outcome was resolution of symptoms rated by the patient as “complete”, “partial” or “unchanged”. Secondary outcome was postoperative headache intensity on the numeric rating scale 0–10.

Data pertaining demographic variables, the surgical procedure, exact location and type of the leak, and preoperative lumbar opening pressure were extracted from the institutional electronic patient data management system. We used a modification of the classification system proposed by Schievink et al. to group CSF leaks into [6]:

- Type 1a: Ventral dural tear with visible CSF egress. These tears are often provoked by a bony microspur penetrating the ventral dura [8].
- Type 1b: Lateral dural leak with visible CSF egress. Often these tears occur in the axilla of the nerve root and are associated with prolapsing arachnoid (meningeal diverticulum) [8]. “Nude” nerve roots with visible CSF egress were classified as type 1b as well [16].
- Type 2: Meningeal diverticulum without visible egress of CSF. Surgical techniques in these cases included ligation of large meningeal diverticula. Cases with a clear dural tear, visible CSF egress and prolapsing arachnoid were classified as type 1b.
- Type 3: CSF-venous fistula.
- Type 4: Indeterminate. This category includes cases with active egress of CSF on surgical exploration, but failure to identify the site of leakage. Surgical techniques in this cases included augmentation of dura.

### Statistics

Statistical analysis was performed using the statistical software SPSS (IBM, Version 25). Descriptive statistics including calculation of the mean and the standard deviation were obtained. We compared matched samples with a Wilcoxon signed-rank test. Two-way comparisons between groups were made with a Mann–Whitney *U* test for continuous variables, and chi-squared or Fisher’s exact test for nominal variables.

We assessed the association between variables and the primary outcome using univariate ordinal logistic regression analysis. Model fitting was assessed using a Likelihood Ratio chi-squared test. Consistency of the data with the fitted model was tested using a Pearson’s chi-squared statistic. Proportional odds assumption was verified using the test of parallel lines. Any variable with a *p* value ≤ 0.15 was integrated into a multivariate model. Association between variables and the secondary outcome was assessed using univariate linear regression analysis. Model fitting was assessed with an *F* statistic in an analysis of variance (ANOVA).

In addition, receiver operating characteristic (ROC) analysis was performed to identify an optimal cut-off point for continuous variables for the prediction of outcome. We used complete resolution of symptoms as a binary outcome variable (complete resolution vs partial/no resolution). Complete resolution was considered a positive result. A cut-off was selected with a low false positive rate and an acceptable sensitivity.

We converted symptom duration to a logarithmic scale, since the data had a right skew. Analyses were done with two-sided tests with a type I error rate of 0.05. Adjustment for multiple comparisons were made using the Bonferroni-Holm method. We addressed missing values first be re-analyzing the source data or in case no value was retrievable by pairwise deletion.

## Results

### Patient population

**Fig. 1:**
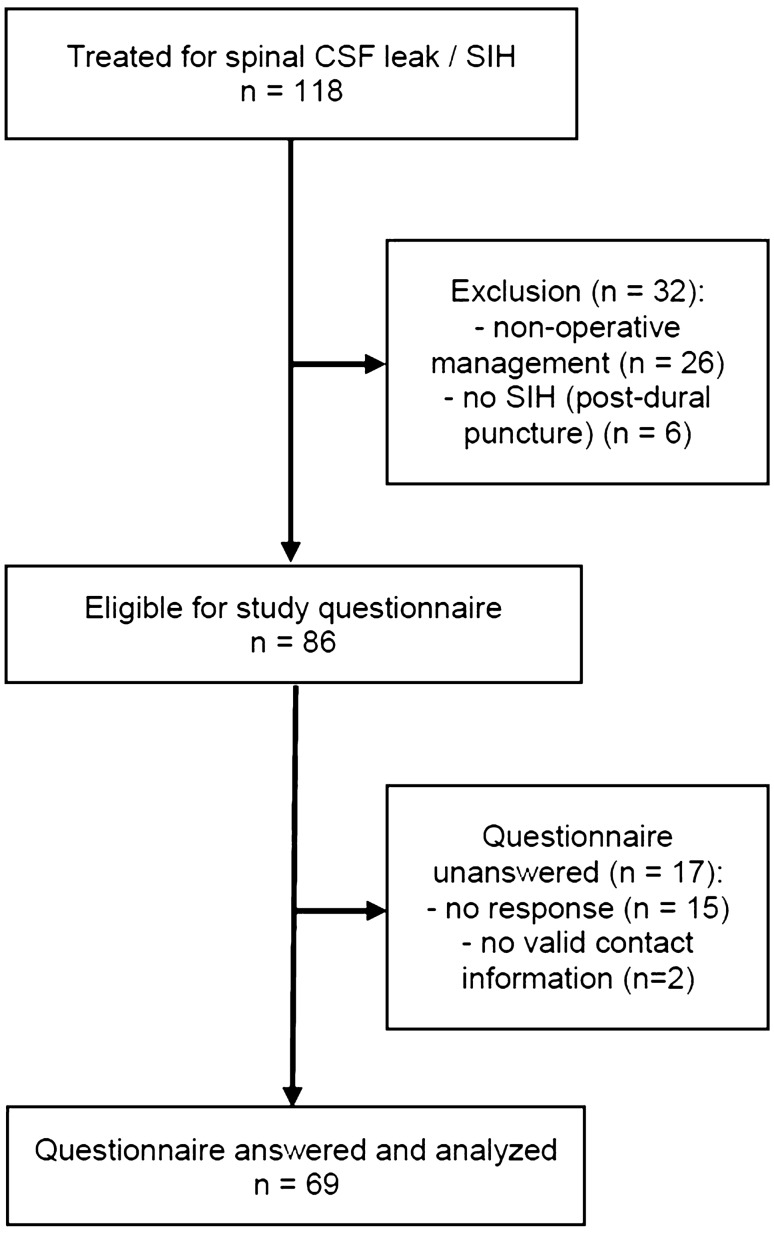
Flowchart displaying patient selection

Between January 2013 and May 2020, we treated 118 patients for SIH or a spinal CSF leak at our institution. Twenty-six of them were managed non-operatively and excluded from the analysis. Six patients were excluded due to a previous lumbar puncture as an initiating event. The remaining 86 patients were treated surgically for SIH and selected for the study. Fifteen of them did not respond to our questionnaire. Additionally, two patients lived abroad and could not be contacted anymore. The remaining 69 (80.2%) returned the questionnaire and were analyzed (Fig. 1).

Mean age was 46.7 years (± 12.1 years) and 47 (68.1%) of the patients were female. Mean duration of follow-up was 2.1 years (± 1.6 years). While 36 (52.2%) of patients reported a complete resolution of symptoms postoperatively, 29 (42.0%) reported only partial resolution with some residual symptoms. Additionally, 4 (5.8%) patients reported no change in symptoms postoperatively. Nevertheless, 65 (94.2%) of patients reported, they would choose surgical treatment again. Mean pre- and postoperative headache intensity on the numeric rating scale were 8.33 (± 1.8) and 1.45 (± 2.0), respectively (*p* < 0.001). Orthostatic headache occurred within five minutes in an upright position in 45 (65.2%) of patients, while 13 (18.8%) reported headache onset after more than five minutes in an upright position and 11 (15.9%) did not report a temporal-positional relationship. Visual and auditory disturbances were reported by 29 (42.0%) and 44 (63.8%) of patients. Mean symptom duration was 355.2 days (± 670.3 days). Twenty-five (36.2%) patients reported an evolution into a less intense or less orthostatic headache character over the course of the disease. Mean lumbar opening pressure was 8.6cmH_2_O (± 6.1cmH_2_O). Fifty-three (76.8%) patients were treated unsuccessfully by lumbar epidural blood patching before undergoing surgery. Postoperatively, 30 (43.5%) patients reported to suffer from transient rebound intracranial hypertension. A ventral dural tear (type 1a) was found 47 (68.1%) patients. A lateral leak (type 1b) was identified in 15 (21.7%) of patients. A meningeal diverticulum without CSF egress (type 2) was considered the culprit lesion in 5 (7.2%) patients. No CSF leak despite the presence of extradural CSF (type 4) was found in 2 (2.9%) patients. We found no CSF-venous fistula in our series (type 3).

The four patients with no change in symptoms postoperatively underwent spinal imaging after surgery (spinal MRI ± CT-myelography), which demonstrated no evidence of a persistent CSF leak in any patient. Among the 29 patients with only partial resolution of symptoms postoperatively, 24 (82.8%) underwent spinal imaging after surgery. In 20 patients, we found no evidence of a persistent leak. In the other four patients, we found an epidural fluid collection. This collection was deemed a postoperative residual rather than a persistent CSF leak due to a clear improvement (albeit not complete resolution) of symptoms, paired with the cranial and spinal imaging findings. In the first patient, the epidural fluid collection regressed and all intracranial imaging signs resolved. In the second patient, typical SIH symptoms clearly improved (while persistent symptoms were attributed to superficial siderosis), but the epidural fluid collection remained constant. In the third patient, the epidural fluid collection regressed. In the fourth patient, intracranial imaging signs resolved despite a constant epidural fluid collection. None of these four patients did undergo dynamic or CT-myelography.

### Predictors of postoperative symptom resolution

**Fig. 2:**
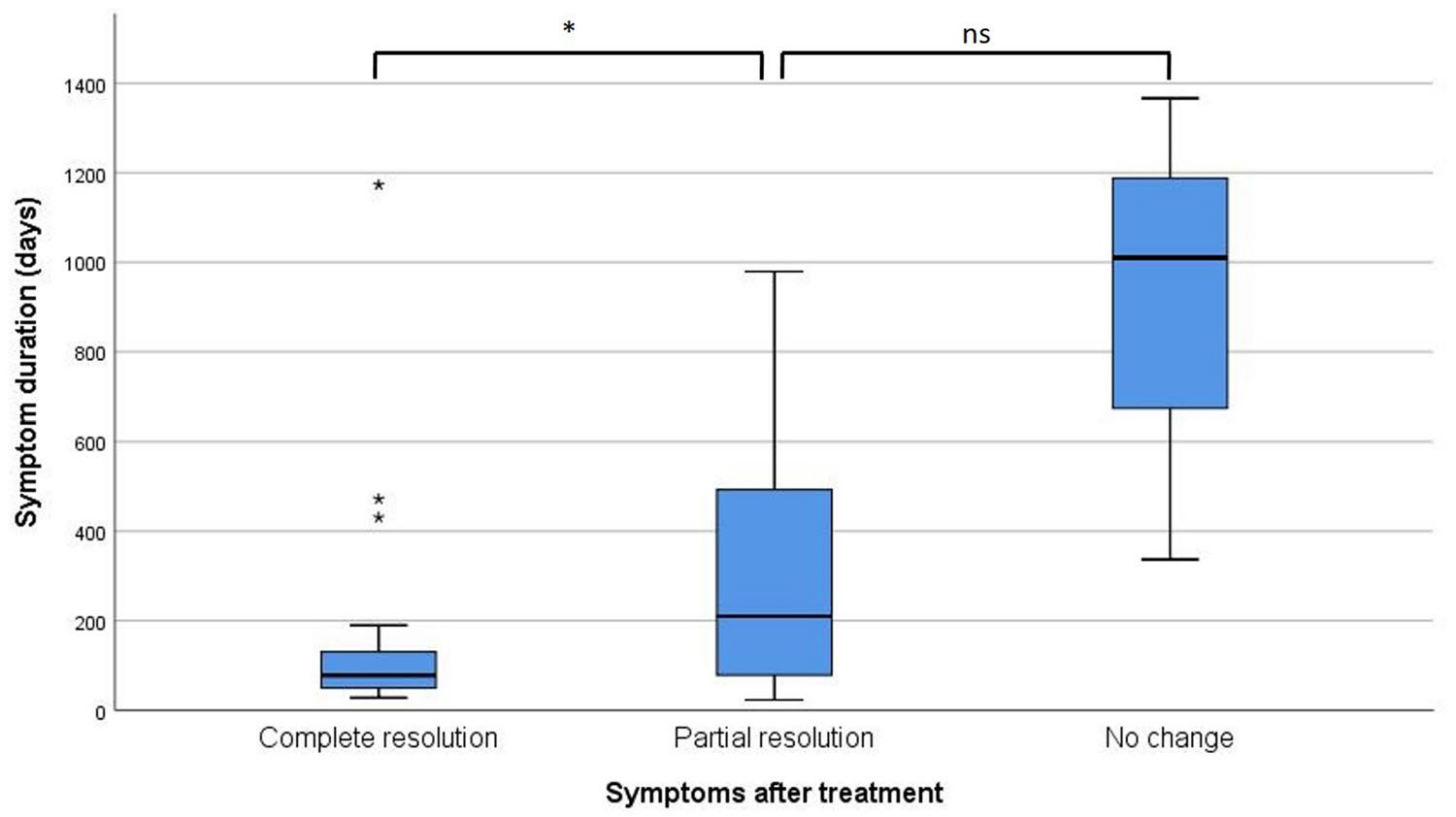
Boxplot displaying symptom duration according to outcome categories. The median is indicated by a horizontal bar. The box covers the interquartile range from the first to the third quartile. Whiskers display minimum and maximum values, whereby outliers (more than 1.5 × interquartile range from first or third quartile) are display separately. *NS* non-significant; *p < 0.05

Using resolution of symptoms as the dependent, ordinal variable, the only significant, predictive model was obtained for preoperative symptom duration (*p* = 0.001) (Table 1), whereby a shorter duration of preoperative symptoms predicted a better outcome. After adjustment for multiple testing, symptom duration remained the only significant predictor of the primary outcome (adjusted alpha-level = 0.004). Two-way comparison between patients with complete symptom resolution and patients with partial resolution yielded significantly shorter symptom duration in the former group (150.2 days vs 524.3 days, *p* = 0.013) (Fig. 2). Patients with partial resolution had a non-significantly shorter symptom duration compared to patients with no change in symptoms (524.3 days vs 904.3 days, *p* = 0.095).

**Table 1:** Predictors of primary outcome

Variable	N/69	*p* value univariat	*p* value multivariat
Age	69/69	0.711	–
Sex	69/69	0.530	–
Preoperative headache intensity (NRS)	67/69	0.059	0.049
Headache ≤ 5 min in upright position	58/69	0.875	–
Visual disturbances	69/69	0.427	–
Auditory disturbances	69/69	0.085	0.112
Evolution into less intense/orthostatic headache	61/69	0.062	0.660
Symptom duration	53/69	0.001	0.013
Lumbar opening pressure (cmH_2_O)	56/69	0.350	–
Preoperative epidural blood patch	69/69	0.744	–
Postoperative rebound hypertension	68/69	0.265	–
Classification of leak (type 1–4)	69/69	0.638	–

*NRS* numeric rating scale

N/69 indicates the number of patients for which data concerning the respective factor was available

Primary outcome was resolution of symptoms rated by the patient as “complete”, “partial” or “no change”

In a multivariate model, symptom duration remained the most powerful and significant predictor (*p* = 0.013) (Table 1). Likelihood ratio chi-squared test revealed a significant improvement in fit of the multivariate model over the null model. Pearson chi-squared test suggested a good fit of the model to the data. Test of parallel lines indicated satisfaction of the assumption of proportional odds.

Interestingly, preoperative headache intensity showed an inverse association with the primary outcome in the multivariate model (*p* = 0.049). Two-way comparison showed a significantly higher preoperative headache intensity in patients with partial symptom resolution compared to patients with no change in symptoms (8.29 vs 5.33, *p* = 0.037). However, in a two-way comparison, no difference in preoperative headache intensity was found between patients with complete symptom resolution and patients with partial resolution (8.61 vs 8.29, *p* = 0.808).

In a post hoc sensitivity analysis using complete symptom resolution as binary, dependent variable (complete symptom resolution vs. partial/no resolution) and non-logarithmic symptom duration in months as explanatory variable, we verified the predictive effect of the latter in a binary logistic regression model (odds ratio 0.920 [0.854–0.992], *p* = 0.029).

### Predictors of postoperative headache intensity

Using postoperative headache intensity as the dependent, continuous variable, symptom duration was the only significant predictor of the secondary outcome (*p* < 0.001; *R* = 0.464) (Table 2). After adjustment for multiple testing, symptom duration remained a significant predictor of the secondary outcome (adjusted alpha-level = 0.004).

**Table 2:** Predictors of secondary outcome

Variable	*p* value univariate	*p* value multivariate
Age	0.237	–
Sex	0.912	–
Preoperative headache intensity (NRS)	0.595	–
Headache ≤ 5 min in upright position	0.616	–
Visual disturbances	0.386	–
Auditory disturbances	0.143	0.356
Evolution into less intense/orthostatic headache	0.296	–
Symptom duration	< 0.001	0.001
Lumbar opening pressure (cmH_2_O)	0.253	–
Preoperative epidural blood patch	0.586	–
Postoperative rebound hypertension	0.945	–
Classification of leak (type 1–4)	0.597	–

*NRS* numeric rating scale

Secondary outcome was postoperative headache intensity rated on the NRS

In a multivariate model, symptom duration remained a significant predictor of the secondary outcome (*p* = 0.001), whereas the presence of auditory disturbances was not predictive (*p* = 0.356) (Table 2). F statistic in ANOVA indicated a good fit of the model.

### Surgical complications

Overall, any surgical complication occurred in 12 patients (17.4%) in our cohort. A new, but transient neurological deficit was found in four patients. In contrast, no patient suffered from a new, persistent neurological deficit postoperatively. Other complications included a recurrent or persistent CSF leak requiring re-operation (*n* = 4), postoperative infection (*n* = 1), postoperative epidural hematoma (*n* = 1), dural adhesion of the myelon (*n* = 1) and intercostal neuralgia (*n* = 1). Re-operation was performed in nine patients (13.0%). Neither the occurrence of complications per se (*p* = 0.302), nor the performance of a re-operation (*p* = 0.154) were associated with the postoperative outcome.

### Definition of a cut-off value

ROC analysis using complete resolution of symptoms as a binary outcome variable and symptom duration as the test variable yielded an area under the curve of 0.734. As a cut-off point with a false positive rate of 0.231 and a sensitivity of 0.556, we selected a duration of 12.4 weeks (87 days) of symptoms. Patients operated with less than 12.4 weeks of symptoms reported complete resolution of symptoms in 71.4%, whereas only 37.5% of patients with more than 12.4 weeks did so (*p* = 0.016).

### Pattern of persistent symptoms

Of 33 patients with only partial or no symptom resolution, 9 (27.3%) reported persistence of orthostatic headache, whereas 21 (63.6%) suffered from persistent non-orthostatic headache. Moreover, 25 (75.8%) reported persistence of accompanying complaints, with the most frequent being cephalic pressure-like sensation (24.0%), acoustic/tinnitus (20%), visual (16%), vertigo (12.0%) and back pain (12.0%).

Data pertaining return to work was available from 63 of all patients. While 30/33 (90.9%) of patients with complete resolution of symptoms reported resumption of work to the full extent as before the disease, only 17/30 (56.7%) of patients with persistent symptoms did so (*p* = 0.003).

## Discussion

Our results demonstrate that a shorter preoperative symptom duration is the most powerful predictor of symptom resolution after surgical treatment of SIH. On univariate analysis, symptom duration was the only significant predictor of the primary and secondary outcome. ROC analysis yielded a preoperative symptom duration of less than 12.4 weeks as the most appropriate cut-off for a better outcome after surgery.

### Outcome after surgical treatment of SIH

In our series, 65 of 69 (94.2%) patients reported at least partial improvement after surgical treatment. Complete resolution of symptoms was achieved in 52.2% of patients. Of note, only refractory cases are referred to surgical repair, which indicates the efficacy of surgery in this difficult to treat patient population. The endpoints of our study are patient reported outcomes. While this represents a strength of our data, one needs to keep in mind that unspecific, unrelated and pre-existing symptoms might interfere. Unspecific symptoms such as dizziness or pre-existing tension type headache might be considered as persistence of symptoms. The clear drop in headache intensity from 8.33 preoperatively to 1.45 postoperatively on the NRS underscores the efficacy of surgery for the treatment of the hallmark symptom of the disease.

Our results are in line with previous results from the literature. Cohen-Gadol et al. reported resolution of symptoms in eight and significant improvement in another 3 out of 13 patients [17]. Schievink et al. reported significant improvement in all of seven patients undergoing surgery for SIH [18]. In a previous series, our group demonstrated improvement of orthostatic symptoms after surgery in 45 of 47 patients [9]. While those studies focused on technical aspects of the procedure, our study focuses on postoperative outcome and its association with preoperative variables. To our knowledge, this study is the first analysis of predictors of outcome after surgical treatment of SIH.

### Timing as predictor of outcome

Preoperative symptom duration was the single most powerful predictor of outcome in our study. Increasing symptom duration was associated with a worse outcome and higher postoperative headache intensity on the NRS on univariate and multivariate analysis. The exact mechanism of this relationship currently remains speculative. One potential explanation for this association can be deduced from data on CSF dynamics in patients with SIH. In our previous work, we demonstrated that the pattern of alteration of CSF dynamics in patients with SIH changes over time [11]. Whereas patients with a short symptom duration display a pathological profile of CSF dynamics, the measures of CSF dynamics change and normalize with increasing symptom duration [11]. This pathophysiological evolution is accompanied by a change in the clinical picture. Patients with long-standing symptoms often presented with atypical, less violent symptoms [11]. Even though the mechanism is unknown, we speculated that this evolution represents a maladaptive change. The pattern of persistent symptoms after surgical treatment with a preponderance of non-orthostatic headache, pressure-like sensation and other atypical complaints indicates the maladaptive nature of these changes. Importantly, the persistence of symptoms through this evolution has a socioeconomic impact with only 56.7% of these patients fully resuming work. It has been suggested, that these changes ensue after a disease duration of 10 weeks. Interestingly, this duration matches the result of our ROC analysis indicating a duration of 12.4 weeks as a cut-off for worse outcome.

Paradoxically at first sight, but in line with the hypothesis mentioned above, more intense headache preoperatively was associated with a better postoperative outcome on multivariate analysis. This likely reflects the more typical and violent presentation of patients, in which the maladaptive process has not occurred yet. Although not reaching statistical significance, patient reported evolution into a less intense or less orthostatic headache character, i.e. patients with a maladaptive process initiated, showed a trend toward worse outcome on univariate analysis. This signal, however, was lost on multivariate analysis, which might be explained by an interaction of this factor with symptom duration, because the occurrence of an evolution of the headache character indicates a longer symptom duration per se.

### Clinical implications

Our results have important implications for the treatment of SIH. While SIH in general is considered a benign and sometimes self-limiting disease [2], our results indicate that expeditious management of patients with persistent symptoms is advantageous. While an initial conservative approach including epidural blood patching is justified, we advocate early surgical repair in patients with persistent symptoms. For the first time, we now propose the specific time frame of 12 weeks from symptom onset until definitive treatment. In case of persistent symptoms despite epidural blood patching, patients should be referred for surgical repair to an experienced team at latest 12 weeks after symptom onset. Although repeated epidural blood patches might have a cumulative effect [19], our results favor early surgical treatment.

## Limitations

Several limitations apply to our analysis. First, this is a single-center study with a limited number of patients that lacks external validation. A part of the data in our study was obtained by a postoperative questionnaire answered by patients on average 2.1 years after treatment. The recall of preoperative pain scores and other symptoms is therefore prone to recall biases. Moreover, we use patient reported outcome measures and it might be difficult to judge for patients, which symptoms are attributable to SIH. Nevertheless, we consider the use of patient reported outcome measures a strength of our data, since the hallmark symptom, headache, is always subjective and cannot be measured on a purely objective scale.

Another limitation is the lack of CSF-venous fistulas in our series. CSF-venous fistulas are increasingly diagnosed and represent an important subgroup of SIH patients. On one hand, the study includes a time before the description of CSF-venous fistulas, on the other hand the lack of CSF-venous fistulas in our series might represent a limitation of the spinal imaging protocol used. Considering the pathophysiological differences between CSF-venous fistulas and conventional dural tears, the influence of preoperative symptom duration on outcome might be different for CSF-venous fistulas. Therefore, our results cannot be extrapolated to this specific patient population. Consequently, the results of our cohort are not generalizable to patients with SIH as a whole and must be interpreted with caution.

## Conclusion

Shorter duration of preoperative symptoms is the most powerful predictor of favorable outcome after surgical treatment of SIH. While an initial attempt of conservative treatment with epidural blood patching is justified, we advocate early surgical treatment not later than 12.4 weeks after onset in case of persistent symptoms and a proven spinal CSF leak.

## Supplementary Information

Below is the link to the electronic supplementary material.

Supplementary file1 (DOCX 35 KB)

## Data Availability

The study data are available and shared at reasonable request of other investigators for purposes of replicating results.
