# Supplementary material for: Outcome after surgical treatment of cerebrospinal fluid leaks in spontaneous intracranial hypotension—a matter of time
Source: J Neurol. 2021 Jul 18;269(3):1439–46. doi: 10.1007/s00415-021-10710-7 (PMC8857147; doi:10.1007/s00415-021-10710-7)
Supplement: Supplementary file 1 — Supplementary file1 (DOCX 35 KB) [file 415_2021_10710_MOESM1_ESM.docx]

**STROBE STATEMENT**

|  | Item No | Recommendation | Page No |  |  |  | Relevant text from manuscript |  |
| --- | --- | --- | --- | --- | --- | --- | --- | --- |
| **Title and abstract** | 1 | (*a*) Indicate the study’s design with a commonly used term in the title or the abstract | 1 |  |  |  |  |  |
|  |  | (*b*) Provide in the abstract an informative and balanced summary of what was done and what was found | 1/2 |  |  |  |  |  |
| Introduction | | |  |  |  |  |  |  |
| Background/rationale | 2 | Explain the scientific background and rationale for the investigation being reported | 3 |  |  |  |  |  |
| Objectives | 3 | State specific objectives, including any prespecified hypotheses | 3 |  |  |  | we hypothesized that a longer duration of preoperative symptoms might negatively affect outcome |  |
| Methods | | |  |  |  |  |  |  |
| Study design | 4 | Present key elements of study design early in the paper | 4 |  |  |  | We conducted a retrospective, observational case-control study. |  |
| Setting | 5 | Describe the setting, locations, and relevant dates, including periods of recruitment, exposure, follow-up, and data collection | 4 |  |  |  | University Hospital of Bern, patients treated between January 2013 and May 2020 |  |
| Participants | 6 | (*a*) Give the eligibility criteria, and the sources and methods of selection of participants. Describe methods of follow-up | 4 |  |  |  | patients treated surgically for SIH at our institution |  |
|  |  | (*b*) For matched studies, give matching criteria and number of exposed and unexposed | N/A |  |  |  |  |  |
| Variables | 7 | Clearly define all outcomes, exposures, predictors, potential confounders, and effect modifiers. Give diagnostic criteria, if applicable | 5 |  |  |  | Primary outcome: resolution of symptoms  Secondary outcomes: headache intensity on the numeric rating scale |  |
| Data sources/ measurement | 8* | For each variable of interest, give sources of data and details of methods of assessment (measurement). Describe comparability of assessment methods if there is more than one group | 5 |  |  |  | Primary and secondary endpoint: questionnaire, patient-reported |  |
| Bias | 9 | Describe any efforts to address potential sources of bias | N/A |  |  |  |  |  |
| Study size | 10 | Explain how the study size was arrived at | N/A |  |  |  |  |  |
| Quantitative variables | 11 | Explain how quantitative variables were handled in the analyses. If applicable, describe which groupings were chosen and why | 6/7 |  |  |  | Association between variables and the secondary outcome was assessed using univariate linear regression analysis. We compared matched samples with a Wilcoxon signed-rank test. Two-way comparisons between groups were made with a Mann-Whitney U test for continuous variables |  |
| Statistical methods | 12 | (*a*) Describe all statistical methods, including those used to control for confounding | 6/7 |  |  |  | We assessed the association between variables and the primary outcome using univariate ordinal logistic regression analysis. Any variable with a p-value ≤ 0.15 was integrated into a multivariate model. Association between variables and the secondary outcome was assessed using univariate linear regression analysis |  |
|  |  | (*b*) Describe any methods used to examine subgroups and interactions | 6/7 |  |  |  | We compared matched samples with a Wilcoxon signed-rank test. Two-way comparisons between groups were made with a Mann-Whitney U test for continuous variables, and Chi-square or Fisher’s exact test for nominal variables. |  |
|  |  | (*c*) Explain how missing data were addressed | 7 |  |  |  | Re-analyzing source data and pairwise deletion |  |
|  |  | (*d*) If applicable, explain how loss to follow-up was addressed | N/A |  |  |  |  |  |
|  |  | (*e*) Describe any sensitivity analyses | 10 |  |  |  | In a post-hoc sensitivity analysis using complete symptom resolution as binary, dependent variable (complete symptom resolution vs. partial/no resolution) and non-logarithmic symptom duration in months as explanatory variable, we verified the predictive effect of the latter in a binary logistic regression model |  |
| Results | | |  |  |  |  |  |  |
| Participants | 13* | (a) Report numbers of individuals at each stage of study—eg numbers potentially eligible, examined for eligibility, confirmed eligible, included in the study, completing follow-up, and analysed | Fig 1, p. 8 |  |  |  | Between January 2013 and May 2020, we treated 118 patients for SIH or a spinal CSF leak at our institution. Twenty-six of them were managed non-operatively and excluded from the analysis. Six patients were excluded due to a previous lumbar puncture as an initiating event. The remaining 86 patients were treated surgically for SIH and selected for the study. Fifteen of them did not respond to our questionnaire. Additionally, two patients lived abroad and could not be contacted anymore. The remaining 69 (80.2%) returned the questionnaire and were analyzed |  |
|  |  | (b) Give reasons for non-participation at each stage | Fig 1, p.8 |  |  |  |  |  |
|  |  | (c) Consider use of a flow diagram | Fig. 1 |  |  |  |  |  |
| Descriptive data | 14* | (a) Give characteristics of study participants (eg demographic, clinical, social) and information on exposures and potential confounders | 8 |  |  |  | Mean age was 46.7 years (+/- 12.1 years) and 47 (68.1%) of the patients were female. |  |
|  |  | (b) Indicate number of participants with missing data for each variable of interest | Table 1 and 2, Fig 1, p.8 |  |  |  |  |  |
|  |  | (c) Summarise follow-up time (eg, average and total amount) | 8 |  |  |  | Mean duration of follow-up was 2.1 years (+/- 1.6 years). |  |
| Outcome data | 15* | Report numbers of outcome events or summary measures over time | 8 |  |  |  | While 36 (52.2%) of patients reported a complete resolution of symptoms postoperatively, 29 (42.0%) reported only partial resolution with some residual symptoms. Additionally, 4 (5.8%) patients reported no change in symptoms postoperatively. |  |
| Main results | 16 | (*a*) Give unadjusted estimates and, if applicable, confounder-adjusted estimates and their precision (eg, 95% confidence interval). Make clear which confounders were adjusted for and why they were included | Table 1 and 2 |  |  |  |  |  |
|  |  | (*b*) Report category boundaries when continuous variables were categorized | N/A |  |  |  |  |  |
|  |  | (*c*) If relevant, consider translating estimates of relative risk into absolute risk for a meaningful time period | N/A |  |  |  |  |  |
| Other analyses | 17 | Report other analyses done—eg analyses of subgroups and interactions, and sensitivity analyses | 10/11 |  |  |  |  |  |
| Discussion | | |  |  |  |  |  |  |
| Key results | 18 | Summarise key results with reference to study objectives | 11 |  |  |  | Our results demonstrate that a shorter preoperative symptom duration is the most powerful predictor of symptom resolution after surgical treatment of SIH |  |
| Limitations | 19 | Discuss limitations of the study, taking into account sources of potential bias or imprecision. Discuss both direction and magnitude of any potential bias | 14 |  |  |  | Retrospective nature, single center, recall biases |  |
| Interpretation | 20 | Give a cautious overall interpretation of results considering objectives, limitations, multiplicity of analyses, results from similar studies, and other relevant evidence | 14 |  |  |  |  |  |
| Generalisability | 21 | Discuss the generalisability (external validity) of the study results | 12 |  |  |  | Our results are in line with previous results from the literature |  |
| Other information | | |  |  |  |  |  |  |
| Funding | 22 | Give the source of funding and the role of the funders for the present study and, if applicable, for the original study on which the present article is based | Title page |  |  |  | No external funding. The study was funded by the Department of Neurosurgery, Inselspital, Bern University Hospital, University of Bern |  |
